# Supplementary material for: A high-resolution mRNA expression time course of embryonic development in zebrafish
Source: eLife. 2017 Nov 16;6:e30860. doi: 10.7554/eLife.30860 (PMC5690287; doi:10.7554/eLife.30860)
Supplement: Supplementary file 6. [file elife-30860-supp6.zip › biolayout-clusters-files/Cluster102-genes.html]

Cluster102


# Cluster102: Genes

| Ensembl ID | Gene Name | Chr | Start | End | Biotype |
| --- | --- | --- | --- | --- | --- |
| ENSDARG00000097055 | ENSDARG00000097055 | 5 | 58446359 | 58532555 | protein\_coding |
| ENSDARG00000091079 | MALSU1 | 16 | 54906505 | 54908666 | protein\_coding |
| ENSDARG00000015502 | adam10b | 25 | 65359 | 75415 | protein\_coding |
| ENSDARG00000037203 | insig2 | 9 | 748316 | 760069 | protein\_coding |
| ENSDARG00000075560 | kmt2cb | 2 | 13164828 | 13354758 | protein\_coding |
| ENSDARG00000078600 | ldlrad3 | 25 | 36754495 | 36760954 | protein\_coding |
| ENSDARG00000099943 | mapre2 | 24 | 35791131 | 35819455 | protein\_coding |
| ENSDARG00000076529 | mn1a | 10 | 4095573 | 4116145 | protein\_coding |
| ENSDARG00000052594 | nkiras2 | 3 | 17717376 | 17722106 | protein\_coding |
| ENSDARG00000102123 | phtf2 | 4 | 223161 | 240969 | protein\_coding |
| ENSDARG00000104542 | ppt2 | 19 | 19274016 | 19282797 | protein\_coding |
| ENSDARG00000053484 | syne2b | 13 | 36949432 | 37128887 | protein\_coding |
| ENSDARG00000008218 | znf410 | 20 | 54559957 | 54570807 | protein\_coding |
